# The Effect of Steps to Promote Higher Levels of Farm Animal Welfare across the EU. Societal *versus* Animal Scientists’ Perceptions of Animal Welfare

**DOI:** 10.3390/ani3030786

**Published:** 2013-08-14

**Authors:** Xavier Averós, Miguel A. Aparicio, Paolo Ferrari, Jonathan H. Guy, Carmen Hubbard, Otto Schmid, Vlatko Ilieski, Hans A. M. Spoolder

**Affiliations:** 1 Departamento de Producción Animal y Ciencia de los Alimentos, Universidad de Extremadura, Avda. de la Universidad s/n Campus Universitario, 10003 Cáceres, Spain; E-Mail: aparicio@unex.es; 2 Centro Ricerche Produzioni Animali C.R.P.A. S.p.A., Viale Timavo 43/2, 42121 Reggio Emilia, Italy; E-Mail: p.ferrari@crpa.it; 3 School of Agriculture, Food and Rural Development, Newcastle University, Newcastle upon Tyne NE1 7RU, UK; E-Mails: jonathan.guy@newcastle.ac.uk (J.H.G.); carmen.hubbard@newcastle.ac.uk (C.H.); 4 Research Institute of Organic Agriculture (FiBL), Ackerstrasse, 5070 Frick, Switzerland; E-Mail: otto.schmid@fibl.org; 5 Faculty of Veterinary Medicine, Ss. Cyril and Methodius University, 1000 Skopje, Republic of Macedonia; E-Mail: vilieski@fvm.ukim.edu.mk; 6 Wageningen UR Livestock Research, P.O. Box 65, 8200 AB Lelystad, The Netherlands; E-Mail: Hans.Spoolder@wur.nl

**Keywords:** animal welfare, European Union, animal welfare initiative, societal perceptions, standards

## Abstract

**Simple Summary:**

We studied different EU production standards and initiatives to determine whether there is still room or not for further animal welfare improvement, and which should be the best way to achieve it. Many of the adopted measures in these standards and initiatives are scientifically supported, but other aspects that are equally important for animal welfare are not included in any of them. Animal welfare improvement should consider, for each country, those aspects actually benefiting animals, but also the social expectations within each country. Economic constraints might explain the gap between what society demands, and what farm animals actually need.

**Abstract:**

Information about animal welfare standards and initiatives from eight European countries was collected, grouped, and compared to EU welfare standards to detect those aspects beyond minimum welfare levels demanded by EU welfare legislation. Literature was reviewed to determine the scientific relevance of standards and initiatives, and those aspects going beyond minimum EU standards. Standards and initiatives were assessed to determine their strengths and weaknesses regarding animal welfare. Attitudes of stakeholders in the improvement of animal welfare were determined through a Policy Delphi exercise. Social perception of animal welfare, economic implications of upraising welfare levels, and differences between countries were considered. Literature review revealed that on-farm space allowance, climate control, and environmental enrichment are relevant for all animal categories. Experts’ assessment revealed that on-farm prevention of thermal stress, air quality, and races and passageways’ design were not sufficiently included. Stakeholders considered that housing conditions are particularly relevant regarding animal welfare, and that animal-based and farm-level indicators are fundamental to monitor the progress of animal welfare. The most notable differences between what society offers and what farm animals are likely to need are related to transportation and space availability, with economic constraints being the most plausible explanation.

## 1. Introduction

The inclusion of animal welfare requirements in European livestock production started in the 1980s, but the issue has undergone a gradual increase in its complexity over recent years [1]. From a practical, technical perspective, the animal welfare concept has evolved from an initial, almost exclusive consideration of the animal, towards a multidimensional concept, which at present has strong, obvious socio-economic implications. It has been, therefore, claimed that overall welfare assessment should involve not only animal welfare science, but also economic and social science aspects [2], as well as moral and ethical considerations [3,4].

In practice, the multidimensionality of animal welfare has been incorporated in most recent EU policies, and is reflected in the EU Strategy for the Protection and Welfare of Animals 2011–2015, the continuation of the EU Action Plan on Animal Welfare 2006–2010 [5]. EU legislation establishes the enforceable minimum welfare thresholds across member states, which are in turn implemented through national legislations. There are also private animal welfare standards and further initiatives, such as quality assurance, organic label schemes, and retailer schemes, that regulate on-farm, transport, and slaughter aspects of livestock production. In many cases, these standards and initiatives include additional requirements beyond the minimum welfare thresholds imposed by EU legislation.

Civil society has played a crucial role in the development of the animal welfare concept, but societal demands are not necessarily associated with consumer attitudes and their purchasing decisions [6]. To make this link stronger, it is first necessary to determine what are the societal/consumer expectations with respect to animal welfare. An additional complexity is to determine to which extent the societal perception of animal welfare matches that of the animal itself. This would help to translate the societal concerns about animal welfare to a number of scientifically sound specific indicators and measures that respond to the demands of consumers and society.

There are many ways in which animal welfare can be improved, but decisions regarding which routes to follow should be made according to their expected impact on society, livestock industry, and the animals themselves [7]. Due to the existing differences among EU countries, decisions should also consider national particularities. The EU-funded project EconWelfare, part of the results from which are described in this paper, aimed to present and analyze the appropriateness of different policy instruments towards higher animal welfare levels, taking into account the concerns of civil society, and the competitiveness of the livestock industry. This paper provides an insight into the most relevant results of EconWelfare regarding the animal. The main aim of this paper is to investigate whether the benefits offered by chain actors through welfare improvement schemes or legislation, address animals’ needs as described in the animal science literature. To achieve this, information relative to different animal welfare standards and initiatives from eight European countries was collected and compared to current EU welfare minimum standards. The animal, civil society, and the livestock production chain dimensions were simultaneously considered. The purpose of this research is to contribute to the debate that surrounds animal welfare, and to provide valuable guidelines for policy makers to further improve it.

## 2. Experimental Section

### 2.1. Animal Welfare Legislation, Standards, and Initiatives in Europe

Different animal welfare standards and initiatives, defined as governmental regulations or private standards and assurance schemes aiming to improve the welfare of farmed animals, were collected in eight European countries (Germany, Spain, Italy, the Netherlands, Poland, Sweden, the United Kingdom, and Macedonia), by means of a standardized on-line survey. The survey was carried out between the end of 2008 and the beginning of 2009, in a commonly manner agreed by all project partners to facilitate systematic investigation. The sources of information for the survey were legislation documents of the different countries, websites of research institutes, animal welfare and consumer NGOs, and interviews with experts. Since it was not possible to define absolute criteria for the relevance of all standards and initiatives, it was decided to ask project partners to select, themselves, five to ten relevant animal welfare initiatives in their country, according to the relevance the animal, society, and supply chain dimensions. The survey asked for general information about different public and private instruments and measures for improved animal welfare, and this was carried out by means of both semi-structured online and structured Excel questionnaires. Information that could not be extracted from the initiatives’ websites was obtained via telephone interview with responsible persons of the initiatives, and by reviewing relevant literature. Specific information from each standard and initiative was then integrated into an Access database. Animal welfare standards and initiatives included organic production standards, non-organic production standards, national legislations, education/training initiatives, research initiatives, quality assurance schemes, and cross-compliance initiatives. Organic production standards were defined according to the EU regulations for organic production (EC Regulation 834/2007 and EC Regulation 889/2008), so that production standards that did not meet any of the requirements were classified as non-organic production standards. National legislations refer to animal welfare legislations from those EU countries that participated in the EconWelfare project. Education/training initiatives refer to specific animal welfare education and training initiatives targeting to different social groups, such as workers from any of the animal production chain steps, school students, and veterinarians. Research initiatives refer to research programs or projects receiving financial support from either the national or the EU level. Quality assurance schemes contemplating animal welfare refer to animal production programs where producers are committed to following a series of animal welfare rules, so that they are periodically audited to ensure that they work accordingly to these rules. Animal welfare cross-compliance initiatives refer to a set of animal welfare requirements that animal producers are required to fulfil in order to receive different direct payments. Specific information about the objectives, implementation, evaluation, and impact of each standard and initiative was collected as well. Initiatives were systematically characterized by all project partners, after a common agreement, according to their goals (animal, chain, farmer, society, or consumer-related goals), instruments (regulatory, labeling, financial/incentives, assurance/guidance, education/information, or development instruments), and actors (farming community, production chain, veterinarians, civil society, public and semi-public, and other private actors) with respect to the animal, the consumer, and society. Additional information about the survey, and the selected standards and initiatives is available elsewhere [8].

### 2.2. Literature Review and Expert Opinion Regarding Key Components of Animal Welfare

In order to support the assessment of the selected standards and initiatives, a review of the scientific literature was undertaken, specifically focusing on those animal welfare aspects of the standards and initiatives going beyond current EU minimum requirements. A review was carried out using the Web of Knowledge online database, using as search terms those distinguishing aspects of the studied standards and initiatives, which were grouped according to the Welfare Quality^®^ principles (*i.e.*, good feeding, good housing, good health, and appropriate behavior) and criteria (*i.e.*, absence of prolonged hunger and thirst; comfort around resting, thermal comfort, ease of movement; absence of injuries, of disease, and of pain induced by management procedures; expression of social and other behaviors, good human-animal relationship, and positive emotional state). The review involved beef cattle, veal calves, dairy cows, sows and piglets, fattening pigs, laying hens and broiler chickens. For each animal category, information relative to on-farm aspects was collected, whereas aspects relative to transport and slaughter were considered across species. Additionally, results obtained in different EU research projects (both national projects within the EU and EU-funded research projects) were assessed. Literature analysis resulted in a list of different, potentially relevant on-farm, transport, and slaughter welfare-related aspects, included in the different standards and initiatives [9,10], which went beyond current EU minimum requirements. This list was discussed with a group of seven invited European animal welfare scientists, most of them being a member or collaborator of Animal Health and Animal Welfare Panel of the European Food Safety Agency (EFSA), during a meeting held in September 2009. The aim of the meeting was to discuss the actual relevance of the mentioned aspects from the animal welfare perspective [11,12]. Animal welfare scientists were presented with the list of potentially relevant aspects, grouped according to the welfare principles and criteria generated by the Welfare Quality^®^ project [13]. Although this is not generally the case, some aspects may be related to more than one criterion. However each aspect was allocated to only one criterion in order to avoid repetitions and to facilitate the comparison of different welfare standards. The experts were then asked to explain their opinion about the effectiveness of each of the aspects of the selected standards and initiatives regarding the animal welfare perspective. 

### 2.3. Stakeholders’ Attitudes to Animal Welfare Policy Instruments and Indicators

The attitudes of stakeholders, different from the aforementioned animal welfare scientists, about how to improve animal welfare were evaluated through a policy survey using the Delphi methodology [14]. A questionnaire was distributed to a range of stakeholders in each of the eight countries participating in EconWelfare. Project partners identified 30–40 national stakeholders, representative of each country, whose expertise covered the animal, supply chain, and society. Five main categories of stakeholders were identified: (1) public authorities (e.g., national, regional or local governments); (2) civil society (e.g., non-governmental organizations and consumer associations); (3) farming communities (e.g., farmers and farmer organizations); (4) other supply chain actors (e.g., retailers, abattoirs, certification and control bodies, food processors and standards setting organizations) and (5) researchers/advisers (e.g., academics and animal welfare researchers, veterinary services). Stakeholders were asked to rate on a Likert scale (an agreement scale) the importance (1 = not important to 5 = very important) of policy objectives and the appropriateness (1 = not appropriate to 5 = very appropriate). and effectiveness (1 = not effective to 5 = very effective) of policy instruments and indicators. The proposed indicators were: (1) animal-based indicators (measures taken on the animal itself); (2) farm-level indicators (measured for the farm as a whole, e.g., housing and management strategies); (3) supply-chain indicators (e.g., labeling and participation/membership of voluntary farm assurance schemes with or without third party inspection and certification); (4) consumer/citizen-based indicators (e.g., changes in awareness of animal welfare issues); and (5) institutional indicators (e.g., levels of monitoring of welfare standards, the number of severe sanctions and the amount of public money spent on research and development). Some demographic variables such as gender and age were also considered. Data collection proceeded in two rounds, between June and November of 2010. Over 450 stakeholders, across all eight countries, were contacted in the first round and almost 200 responses were received by the end of July 2010. In the second round, the respondents of the first round received a summary of the findings and their initial responses, and were given the opportunity to revise their responses. Potential effects of the stakeholder category in the ratings were tested by means of one-way ANOVA’s (*P* < 0.05). In case of statistically significant effect, LSD *post hoc* means separations were obtained.

### 2.4. Animal Welfare Scientists’ Assessment of Legislation, Standards, and Initiatives

During the September 2009 meeting, animal welfare scientists assessed the advantages and disadvantages of each of the different standards and initiatives for welfare. Analysis was undertaken according to the availability of comparable information on those welfare aspects going beyond current minimum EU requirements. The analysis was based on the animal welfare scientists’ opinion about the perceived benefits for animal welfare of the considered standards and initiatives on farm, during transport, and at the slaughterhouse. Animal welfare scientists were asked to explain their opinion about the effectiveness of each upgraded standard and initiative and to list, for each category of farmed animal, the most important, distinguishing aspects of the upgraded standards and initiatives that would benefit animal welfare beyond EU baseline legislation. For each category of farmed animal, experts were asked to take into account:

- The requirements of the baseline EU legislation on animal welfare.
- The housing and rearing systems commonly used in the EU.
- The scientific knowledge about the capability of the upgraded standards and initiatives to improve animal welfare.

## 3. Results and Discussion

### 3.1. Animal Welfare Legislation, Standards, and Initiatives in Europe

Nowadays, animal welfare is a clear matter of societal concern and debate [15], and this is reflected in the number of standards and initiatives (41 regulatory standards and initiatives consisting of 8 organic standards, 26 non-organic standards and 7 national legislations; and 39 non-regulatory initiatives consisting of 29 education/information, 5 research, 3 quality assurance schemes, and 2 cross-compliance initiatives) which were collected and analyzed, despite animal welfare not being the main objective of some of them [8]. On-farm, transport, and slaughter aspects found in private standards and initiatives, which go beyond EU baseline legislation are listed in Table 1. For ease of interpretation, only those aspects being scored at least five times across standards and initiatives and relating to more than two countries are shown in the table. Some on-farm aspects were regulated in all species and countries, particularly those relative to space allowance/stocking density, feeding and drinking, and the use of litter/bedding material/enrichment. Important aspects for all species during transport were prohibiting the use of sedatives/tranquilizers, animals being fit for transport, adequate loading facilities and handling procedures, the provision of bedding for younger animals, and the restriction of journey duration. Important aspects for slaughterhouses were the improvement of facilities and handling practices during the unloading, lairage, and slaughter, as well as staff education. Some similarities were found for swine and cattle species, particularly in relation to additional on-farm space allowance, restrictions in the use of slatted floors, roughage provision, castration and other surgical practices in beef cattle and growing-finishing pigs. This might be due to the existing similarities between both types of production systems.

**Table 1:** Summary of the main aspects, found in at least five private standards and initiatives, with requirements beyond EU legislation, for each species on-farm, during transport, and at slaughter (source: [8]).

**On farm,** **cattle**	Tethering restricted, more space and light requirements, slatted floors forbidden or limited, specific bedding requirements, stable groups to avoid aggressive behaviors, outdoor access, more specific feeding requirements (e.g., roughage), longer weaning periods, provision of calving pens, adequate anaesthesia for castration, non-allowance of certain surgical practices
**On farm,** **pigs**	Availability of litter, slatted floors forbidden or restricted, possibilities for investigation and manipulating activities, provision of roughage, no hormonal treatments, adequate anaesthesia for castration, limitation of certain surgical practices, more space allowance
**On farm,** **poultry**	More light requirements, more perches and nests, access to dust baths, better management of litter materials, outdoor run and pasture, lower indoor and outdoor stocking densities, better access to fresh water, restrictions in breeding (mainly broilers), higher frequency of regular visits
**Transport**	Interdiction of sedatives/tranquilizers (not allowed in organic husbandry), provision of bedding material for the youngest in transport vehicles, more drinking, resting and feeding possibilities before transport, adequate pathway/ramps design, the separation of unfamiliar groups, reduced length of journey
**Slaughter**	More lairage requirements (start of lairage, space, lighting, floors *etc.*), the avoidance of group mixing, electric stimulation prohibited, time between stunning and bleeding, specific education of the staff

Aspects found in at least five standards and in at least two countries.

Differences in standards and initiatives were detected among the eight studied countries, with some of them having national legislation with additional requirements beyond EU animal welfare legislation. In this sense, Sweden has stricter regulations for the housing and management of cattle, pigs, poultry, and the transportation of livestock, while Germany has additional requirements for pigs and poultry space allowance, and for transportation and slaughter. The UK has additional requirements for transport and slaughter, and for space allowance of pigs. The Netherlands has additional requirements regarding the housing and management of pigs, emphasizing space allowance, mutilations, use of solid floors, aspects relative to group housing in sows, and aspects relative to mixing and feeder space in growing-finishing pigs.

### 3.2. Literature Review and Animal Welfare Scientists’ Opinion of Key Components of Animal Welfare

Table 2 summarizes those on-farm aspects of the selected standards and initiatives going beyond legislation, classified according to Welfare Quality^®^ principles and criteria. For the first principle of good feeding, emphasis was placed on supplying roughage to ruminants. For these species, a sufficient quantity of high quality roughage reduces the prevalence of abnormal behaviors, and promotes rumen development in veal calves [16,17], reduces the risk of gastrointestinal disorders in beef cattle [18] and prevents metabolic disorders in dairy cows [19].

**Table 2:** On-farm aspects included in the different standards and initiatives that were considered as relevant for animal welfare by the consulted animal welfare scientists (source: [12]).

Welfare Quality Principles	Welfare Quality Criteria	Distinguishing aspects in initiatives studied as part of the EconWelfare project	Beef cattle	Veal calves	Dairy cows	Sows and piglets	Fattening pigs	Laying Hens	Broiler chickens
**Good feeding**	Absence of prolonged hunger	Allowance of roughage	×	×	×				
		Facilities to avoid competition for feed			×		×		
		Minimum age at weaning				×			
	Absence of prolonged thirst	Facilities to avoid competition for water			×		×		
**Good housing**	Comfort around resting	Bedding Material			×				×
	Thermal comfort	Microclimate control	×	×		×	×	×	×
		Air quality (toxic gases, dust)		×					
	Ease of movement	Avoidance of tethering/individual housing			×	×			
		Space allowance	×	×	×		×	×	×
**Good health**	Absence of injuries	Avoidance/limitation of slatted floors				×	×		
	Absence of disease	Restricted use of antibiotics		×					
		Avoidance hyper muscled/fast growing breeds							×
	Absence of pain induced by management procedures	Avoidance of electric prods/trainers			×				
		Avoidance of mutilations	×					×	
**Appropriate behavior**	Expression of social behavior	Stable groups to avoid aggressive behavior		×		×			
	Expression of other behavior	Environmental enrichment				×		×	
	Good human-animal relationship	Regular visits						×	×

For good housing, animal welfare scientists stressed the importance of microclimate control and space allowance as relevant aspects for most of the species and categories of farmed animals. Environmental control is basic when climate conditions are extreme. In this sense, heat stress may be detrimental for the reproductive performance of pregnant sows [20], the behavior and performance of growing-finishing pigs [21,22], the immune function, productive performance, and mortality of laying hens [23], and the health and performance of broilers [24].

Increasing the space allowance would, in its turn, reduce the prevalence of gastrointestinal disorders in veal calves and beef cattle [18,25], result in cleaner animals and in a lower prevalence of mastitis in cows housed in straw yards, and reduce the occurrence of agonistic behaviors and increase the resting time in cows housed in cubicles [26,27]. Higher space allowances would also benefit the lying behavior and the growth of growing-finishing pigs [28,29]. In broiler chickens, lower stocking densities would increase heat dissipation and enhance growth rate, reducing the occurrence of dermatitis when litter conditions are inadequate [30]. In laying hens, greater space allowance would allow the expression of a wider behavioral repertoire, although some behaviors, such as cannibalism, are extremely negative for other individuals [31].

Not only the quantity, but also the quality of space is important. Floor quality is linked to the prevalence of leg problems, and key aspects to reduce aggression would be the provision of sufficient space when mixing unfamiliar sows and the improvement of floor characteristics through the use of bedding [32,33]. Feeder characteristics that minimize the monopolization of feeder by dominant sows are also important to reduce the occurrence of aggressions at the feeder [33]. The use of bedding in the lying area is also positive for dairy cows, although care must be taken when choosing the bedding material, in order to avoid lesions and microbial development [34]. Although all floor types have advantages and disadvantages, slatted floors increase the prevalence of injuries and lameness, having negative implications for welfare [29,35,36]. Partially slatted floors have been proposed as a practical compromise to solve this problem [35].

The third principle of good health focused on its relationship with housing and management procedures. Poor ventilation is one of the main causes of Bovine Respiratory Disease in calves [37]. This disease is routinely treated with antibiotics, although this may have undesired side effects such as kidney lesions [38].

With respect to mutilations, castration is an obvious painful procedure for young bulls [39] that should not be performed routinely [40]. In the case of laying hens, beak trimming has been found to reduce food consumption and egg weight, despite reducing the occurrence of cannibalism [41]. The use of genetically improved animal breeds has a number of performance advantages, although this has also resulted in the occurrence of different welfare problems. Fast growth in broilers is often associated with reduced physical activity, and consequently to extremities weakness [30]. Therefore, the use of slow growing breeds and a reduction in stocking density might contribute to improved welfare of broiler chickens [30].

Regarding the ability to perform an appropriate behavioral repertoire, group housing has beneficial effects on the welfare of veal calves once the group is stable [42], reducing the inactivity time, promoting social interactions [43], and improving weight gain and feed efficiency [44]. Dairy cows are strongly motivated for locomotion [45], and movement restriction may thwart this movement motivation, and compromise other social motivations. According to behavioral and hormonal measurements, social isolation has negative implications for dairy cows [46]. Standing rumination is an indicator of discomfort, and it has been found that the percentage of cows lying down while ruminating decreased, during indoor tethering compared to the same cows when they were grazing outdoors [47]. Group housing would consequently be positive for cows’ welfare, although care must be taken in order to minimize competition for resources [48]. In this sense, measures to minimize competition for resources, and particularly food, may also be positive for growing-finishing pigs [49,50]. According to [33], unfavorable social, management and climate conditions may cause chronic stress, low feed intake, and reduce the reproductive performance of group-housed, pregnant sows.

The provision of dust baths and perches to laying hens may reduce levels of frustration and stress, and thus the occurrence of welfare problems [31,51]. Nevertheless, the use of perches has also been associated with cannibalism [52]. Enrichment methods are also positive for broilers, provided that they stimulate their physical activity [30].

Early weaning is associated with behavioral problems, and induces a stress reaction in piglets, and so weaning before 21 days of age would not be recommended [53]. A positive human-animal relationship is beneficial for poultry, and generally for all farmed species [54]. Regular visits to detect and solve problems will therefore largely contribute to the improvement of the welfare of animals, provided that the attitude towards animals of the involved persons is positive. In comparison to on-farm animal welfare, the number of aspects that were identified by animal welfare scientists as important for the welfare of animals during transport and slaughter was relatively small (Table 3). Although the appropriate design of ramps and passageways, and stunning efficiency were relevant for most species, bovine species had a comparatively higher number of aspects going beyond current EU legislation.

**Table 3:** Transport and slaughter aspects included in the different standards and initiatives considered as relevant for animal welfare by the consulted animal welfare scientists (source: [12]).

Welfare Quality Principles	Welfare Quality Criteria	Distinguishing aspects	Beef cattle	Veal calves	Dairy cows	Sows and piglets	Fattening pigs	Laying Hens	Broiler chickens
**Good feeding**	Absence of prolonged thirst	Drinking before loading	×	×	×				
**Good housing**	Ease of movement	Race and passageways design (including ramps)	×	×	×	×	×		
**Good health**	Absence of pain induced by management procedures	Avoidance of electric prods at loading/unloading	×	×	×				
		Stunning efficiency at slaughter	×	×	×	×	×	×	×
**Appropriate behavior**	Expression of social behavior	Avoidance of mixing during transport/slaughter				×	×		

Water deprivation may cause dehydration and weight loss in animals, particularly during long distance transport [55]. The rumen acts a water reservoir [56], so that the administration of either drinking water or electrolyte solutions would be advisable to adequately prepare cattle for transport [57]. Loading and unloading can be stressful procedures for cattle and pigs so that lairage pens, races, and passageways to gain access to the truck and the truck’s loading systems should be designed according to the particularities and needs of each species [58,59,60,61]. The use of electric prods is very detrimental for cattle [62], so that its use has been proposed to be restricted only to those cases where the animal refuses to move [63]. Mixing unknown pigs during transport and lairage will almost unavoidably cause fighting, generating welfare problems that may lead to increased mortality at the end of the journey and meat and carcass problems [64,65]. These facts would support the implementation of these aspects within the studied standards and initiatives. It is surprising, nevertheless, the lack of agreement between scientific knowledge and some aspects of initiatives and standards, such as is the case of the control of ambient conditions during transport. Transport is a stressful experience for animals, with journey duration being a largely contributing factor [55], although it is also known that, under certain transport conditions, climate conditions may be largely more detrimental than the journey duration itself [66,67]. Nevertheless, hardly any mention of climate control measures during transportation was found in any of the standards and initiatives, although some of them have specific requirements on journey duration. This might be the consequence of scientific information being produced after the creation of standards and initiatives, and would highlight the importance of continuously checking all welfare standards, both at the public and private levels, and eventually updating them according to the latest scientific advances. Slaughter is a critical moment for pigs, cattle, and poultry, and therefore stunning methods should ensure their unconsciousness [63,68,69].

### 3.3. Stakeholders’ Attitudes to Animal Welfare Policies

The Delphi results presented in this section have already been partly presented elsewhere [70,71]. From the 458 stakeholders that were initially invited, 197 (43%) responded to the two Delphi rounds. Macedonia, Spain, United Kingdom, Poland, Italy, Germany, Sweden, and Netherlands accounted for 26.9%, 16.8%, 15.2%, 11.2%, 8.6%, 8.1%, 8.1%, and 5.1% of the responses, respectively. According to stakeholder category, researchers/advisers, other supply chain actors, civil society, public authorities, and farming community accounted for 28%, 28%, 18%, 16%, and 10% of responses, respectively. For beef cattle, dairy cows, and sows and piglets, the mean number of animal welfare aspects, contained within each of the main topics, that were cited as important by consumers and non-governmental organizations (NGOs) are shown in Table 4.

**Table 4:** Average (±standard error) number of issues, for beef cattle, dairy cows, and sows, and piglets, contained within each of the main animal welfare topics, that were cited as important by consumers and non-governmental organizations (NGOs) within the studied countries^1^.

	Beef Cattle		Dairy Cows		Sows and piglets	
	Consumers	NGOs	Consumers	NGOs	Consumers	NGOs
**Housing**	1.3 ± 0.7	2.0 ± 0.7	0.4 ± 0.3	4.3 ± 0.9	0	1.9 ± 0.5
**Feeding**	0.4 ± 0.2	0.4 ± 0.3	0.1 ± 0.1	0.4 ± 0.2	–	–
**Natural behavior**	0.1 ± 0.1	1.0 ± 0.3	0.1 ± 0.1	1.7 ± 0.4	0	2.1 ± 0.7
**Hygiene/health**	0.6 ± 0.6	0.3 ± 0.2	0.4 ± 0.4	2.0 ± 0.7	0	0.3 ± 0.2
**Transport**	0.3 ± 0.2	1.1 ± 0.7	0.1 ± 0.1	0.6 ± 0.3	–	–
**Slaughter**	0.6 ± 0.3	0.4 ± 0.3	0.3 ± 0.2	0	–	–

^1^ Italy, The Netherlands, Sweden, Germany, Poland, the United Kingdom, Spain.

The low number of issues that were considered as important by both consumers and NGOs is particularly remarkable. This might be due to the fact that, despite the great importance of animal welfare for both stakeholder groups, their practical knowledge of the actual issues is limited. NGOs cited on average a higher number of animal welfare aspects than consumers, although this fact was dependent on the country. For instance, Swedish consumers cited a higher number of important aspects for beef cattle than did Swedish NGOs, while Dutch and Spanish consumers and NGOs found that hardly any of the proposed issues were important for animal welfare. The importance given to each topic depended on the species, although it was generally agreed that housing was the topic with more important aspects. This would suggest that measures aiming to improve animal housing conditions would have a good social acceptation and that, in most of the sampled countries, there is still some way to go before stakeholders would be satisfied with the levels of farm animal welfare. Current society perception about animal production is complex [72], due to the existence of underlying cultural aspects that make this perception different from one individual to another [73,74]. Consequently, it is not easy to disentangle how society perceives and understands animal welfare, with good examples showing how consumers associate, for instance, aspects relative to the processing of meat with improved on-farm welfare conditions [75].

Previous studies suggest some level of disagreement between different stakeholder groups regarding the animal welfare subject [76,77]. Nevertheless, as shown in Table 5, a general agreement was found in the perception of both animal-based and farm-level indicators as the most effective means to monitor animal welfare by all stakeholder groups. For ‘animal-based’ there were differences between groups (*P* < 0.05), with farming community scoring lower, as opposed to researchers/advisers and, to a lesser extent, civil society. All groups also agreed that animal behavior and animal health are the most effective animal-based indicators, with researchers/advisers scoring the highest means in the case of animal behavior (*P* < 0.05; Table 6).

Differences across groups are particularly significant as regards ‘farm-level indicators’ (*P* < 0.05 in practically all cases; Table 7), with civil society rating all indicators but ‘indicators related to the use of hormones/growth promoters’ above 4. Civil society ranked top ‘indicators related to management strategies for minimizing pain’, followed by ‘indicators related to space and ventilation’, ‘housing design and bedding material’, and ‘health care programs’. Researchers/advisers also rated high these indicators. Nonetheless, the latter group found that providing animals with ‘natural feeding/grazing and outdoor access’ was a less important aspect, while civil society rated it among the most important aspects. Some differences were detected between countries, with The Netherlands scoring ‘indicators related to management strategies for minimizing pain’ as the most important and Germany the second most important, while Poland and Sweden ranked ‘indicators related to natural feeding/grazing and outdoor access’ as the most effective. Nevertheless, all countries scored ‘indicators related to space and ventilation’ amongst the top two measures. Differences between countries regarding the perceived effectiveness of farm-based indicators to monitor animal welfare might be due to a combination of different aspects, such as cultural differences, different levels of animal welfare awareness, or differences in the animal welfare perception according to regional differences in the characteristics of production systems.

**Table 5:** Rating (1 to 5 scale ^1^) of the effectiveness of broad categories of indicators according to the different Expert groups (means) (source: [71]).

*Indicators*	*Public authorities*	*Civil society*	*Farming community*	*Chain actors*	*Researchers/* *advisers*	*P-value* *(F-test)*
**Animal-based **	4.3 ^abc^	4.6 ^ab^	4.1 ^c^	4.3 ^b^	4.6 ^a^	0.016
**Farm-level **	4.3	4.3	3.8	4.1	4.1	0.100
**Supply-chain **	3.6	3.7	3.3	3.6	3.6	0.599
**Consumer-based**	3.3	3.6	2.8	3.6	3.4	0.061
**Institutional **	3.2 ^ab^	3.5 ^a^	2.8 ^b^	3.5 ^a^	3.1 ^ab^	0.048

^1^ 1 = the lowest effectiveness, to 5 = the highest effectiveness; ^a,b,c^ within a row, different letters indicate statistically significant differences (*P* < 0.05).

**Table 6:** Rating (1 to 5 scale ^1^) of the effectiveness of ‘Animal–based’ indicators according to each Expert category (means) (source: [71]).

*Indicators of*	*Public authorities*	*Civil society*	*Farming community*	*Chain actors*	*Researchers/* *advisers*	*P-value* *(F-test)*
**Animal behavior**	4.4 ^ab^	4.5 ^ab^	4.1 ^bc^	4.1 ^c^	4.5 ^a^	0.020
**Animal health **	4.2	4.7	4.4	4.4	4.3	0.196
**How the animal responds to how it is fed**	3.5	3.7	3.6	3.7	3.4	0.627
**How the animal responds to how it is housed **	4.2	4.3	3.7	4.1	4.0	0.219

^1^ 1 = the lowest effectiveness, to 5 = the highest effectiveness; ^a,b,c^ within a row, different letters indicate statistically significant differences (*P* < 0.05).

**Table 7:** Rating (1 to 5 scale ^1^) of the effectiveness of farm-level indicators according to each Expert category (means) (source: [71]).

*Indicators related to*	*Public authorities*	*Civil society*	*Farming community*	*Chain actors*	*Researchers/* *advisers*	*P-value* *(F-test)*
**Space & ventilation**	4.1 ^b^	4.5 ^a^	3.9 ^c^	4.4 ^ab^	4.1 ^bc^	0.017
**Housing design & bedding material**	4.3 ^ab^	4.5 ^a^	3.5 ^c^	4.2 ^b^	4.0 ^b^	0.001
**Access to natural feeding/** **grazing & outdoor**	4.0 ^ab^	4.5 ^a^	3.2 ^c^	4.0 ^b^	3.9 ^b^	0.001
**Use of hormones/growth promoters**	3.1	3.6	2.8	3.8	3.2	0.062
**Breeding strategies**	3.6 ^ab^	4.1 ^a^	3.1 ^c^	3.9 ^ab^	3.4 ^bc^	0.009
**Health care programmes**	3.9 ^c^	4.5 ^a^	4.0 ^bc^	4.3 ^ab^	4.0 ^bc^	0.018
**Management strategies for minimizing pain**	4.0 ^b^	4.7 ^a^	3.2 ^c^	4.0 ^b^	4.0 ^b^	0.000

^1^ 1 = the lowest effectiveness, to 5 = the highest effectiveness; ^a,b,c^ within a row, different letters indicate statistically significant differences (*P* < 0.05).

The general agreement found between researchers/advisers and civil society on the effectiveness of the different indicators to monitor animal welfare suggests some degree of agreement between the societal perception on the subject, and those aspects scientifically relevant for animals, as already indicated by other authors [78,79]. This is important, since scientific knowledge should form the basis of measures to improve animal welfare, and have the real support of consumers and society. According to the results of the Delphi survey, if higher levels of farm animal welfare standards are to be achieved in the future, the four proposed policy objectives should be considered.

The results of the Delphi survey showed that researchers/advisers and civil society also agreed in that the use of a combination of animal-based and farm-level indicators would be a suitable strategy to monitor the welfare conditions of farmed animals. From all the production aspects, enhancing housing conditions was perceived as an effective way to globally improve animal welfare. In this sense, societal concern about space restriction and floor characteristics is relatively high compared to other welfare aspects [80,81,82], suggesting that measures to improve welfare in this direction might have a good social acceptation, although education and outreach activities appear to be a prerequisite to raise social awareness of the efforts made towards the upgrading of welfare conditions. In this sense, results also revealed that education of both general public and production chain is essential to improve animal welfare. Due to both its importance for society and its tangibility, space allowance might be an important issue to base any sectorial effort aiming to improve social awareness and the industry image. On the other hand, the Delphi survey revealed the perception of civil society about the role of government as the guardian for implementing higher animal welfare standards, as opposed to the perception of researchers/advisers who would prefer a ‘carrot’ rather than a ‘stick’ for improving both on- and off-farm animal welfare standards. Social perception of animal welfare varied according to the species (as shown in the examples given in Table 3) and country. The latter agrees with [82] in relation to the different attitudes of European citizens towards pig production systems.

Although farm and animal-related welfare indicators are important in the monitoring of the improvement of animal welfare, the latter was more relevant according to the opinion of scientists/advisers and society (*i.e.*, scored higher). Nevertheless, it is widely accepted that system measures are not fully reliable predictors of welfare and that measures should be mainly outcome-based, with input measures being used only if they are clearly related to outcome measures [83]. The use of animal-based indicators to monitor animal welfare is gaining gradual acceptance [63], and a combination of individual and system measurements might be considered as the best approach to monitor and improve the welfare of livestock species [84]. The Delphi results are also strengthening these findings, although it should be considered that scores for the appropriateness of a policy instrument for achieving a certain policy objective are likely to depend both on the experiences of the policy instrument within a country and the background and training of the individuals within their particular interest groups.

### 3.4. Animal Welfare Scientists’ Assessment of Legislation, Standards, and Initiatives

Table 8 shows, for each category of farmed animal, the percentage of initiatives including aspects beyond current EU legislation. Animal welfare scientists considered stunning efficiency at slaughter as an important aspect in all species, despite none of the initiatives for veal calves including it. Animal welfare scientists considered on-farm space allowance as an important aspect for fattening pigs, but less so for sows and piglets. For most of the initiatives (80.3% on average across species/animal category), space requirements went, to some extent, beyond the EU legislation (including sows and piglets). Animal welfare scientists also considered on-farm prevention of cold and heat stress, air quality, and design of races and passageways, to be important aspects for most categories of farmed animals, although in both cases the percentage of initiatives including them was relatively low. On-farm provision of roughage was important for bovine species, with 91.1% of initiatives including it in their production standards. Different aspects were important for only one or a few animal categories, although a majority of the affected initiatives included it. This was the case for the use of antibiotics (100% of initiatives), avoidance of fast-growing breeds (66.7% of initiatives), prohibition of mutilations (65.9% of initiatives), and provision of group housing/absence of tethering (58.9% of initiatives).

**Table 8:** Percentage of standards and initiatives that include aspects that go beyond current EU legislation, and that are relevant for the welfare of each species/category of farmed animals (according to expert assessment using the Welfare Quality^®^ principles).

	Veal Calves (6)	Beef Cattle (11)	Dairy Cows (15)	Sows and piglets (15)	Fattening Pigs (13)	Laying Hens (14)	Broilers (12)
***On farm aspects***							
Allowance of roughage/fibre	100	100	73.3	–	–	–	–
Prevention of cold/heat stress and air quality	–	18.2	–	26.7	46.2	21.4	8.3
Space allowance	100	90.9	66.7	–	84.6	64.3	75
Mutilations	–	81.8	–	–	–	50	–
Restricted use of antibiotics	100	–	–	–	–	–	–
Group housing/avoidance of tethering	50	–	66.7	60	–	–	–
Stable groups to avoid aggressive behavior	–	–	–	13.3	–	–	–
Facilities to avoid competition for feed	–	–	26.7	–	46.2	–	–
Facilities to avoid competition for water	–	–	20	–	30.8	–	–
Bedding material/Enrichment	–	–	73.3	73.3	–	–	41.7
Minimum age at weaning	–	–	–	53.3	–	–	–
Avoidance or limitation of slatted floors	–	–	–	33.3	92.3	–	–
Availability of dust bath	–	–	–	–	–	42.9	–
Regular visits/inspections by stockperson	–	–	–	–	–	14.3	25
Avoidance of fast-growing/hyper muscled breeds	–	–	–	–	–	–	66.7
***During transport and slaughter aspects***							
Drinking before loading on vehicles for transport	16.7	18.2	13.3	–	–	–	–
Avoidance of electric prods	33.3	90.9	73.3	–	–	–	–
Race and passageways design	0	36.4	20	13.3	76.9	–	–
Stunning efficiency	0	45.5	33.3	33.3	53.8	35.7	25
Separation of unfamiliar groups at transport/slaughter	–	–	–	33.3	61.5	–	–

Between brackets: number of standards and initiatives for each species/category of farmed animals.

The general implementation of some of the measures already contemplated in the studied standards and initiatives would improve the welfare of farmed species, although some impact on the production costs might realistically be expected [85]. For instance, reaching optimal space allowances for animal welfare would imply an increase in production costs, so that some reduction of farm incomes should be expected. Additionally, differences in the space requirements amongst EU countries would make this economic impact largely dependent on these existing regional differences. The impact of upgrading welfare standards should also be expected to depend on the species, since different measures should be applied for each of them. These are important aspects to bear in mind if welfare standards are to be upgraded, and are discussed more comprehensively in another paper related to the EconWelfare project [86].

## 4. Conclusions

Steps to improve animal welfare in the EU should be both animal- and system-oriented, and scientifically based. They should take into account species specific needs, but also society expectations in order to maximize the acceptation of animal products by consumers. In both cases there is a strong regional component. The different studied initiatives may inspire further upgrading of current EU welfare standards, since a good number of the adopted measures are supported by scientific evidence. Nevertheless, science also supports other aspects that are equally important for animal welfare, but are not included in any of the studied standards and initiatives. Routes towards the effective improvement of animal welfare should simultaneously take into account, for each country, species-specific aspects implying a real benefit for animals, but also the specific needs/demands on animal welfare within the society of each country. The EconWelfare data suggest that both appear to go well together, although some aspects of welfare improvement are poorly addressed in current standards and legislation. Most notable issues in this respect are those related to animal transportation and the quality of space in intensive husbandry systems. Economic constraints are the most obvious explanation for this difference between what society offers, and what farm animals are likely to need for their own welfare.
